# A novel diffuse gastric cancer susceptibility variant in E-cadherin (*CDH1*) intron 2: A case control study in an Italian population

**DOI:** 10.1186/1471-2407-8-138

**Published:** 2008-05-15

**Authors:** Soroush Nasri, Helen More, Francesco Graziano, Annamaria Ruzzo, Emily Wilson, Anita Dunbier, Cushla McKinney, Tony Merriman, Parry Guilford, Mauro Magnani, Bostjan Humar

**Affiliations:** 1 Cancer Genetics Laboratory, Department of Biochemistry, University of Otago, Dunedin 9054, Aotearoa New Zealand; 2 Medical Oncology Unit, Azienda Ospedale "S. Salvatore", 61100 Pesaro, Italy; 3 Institute of Biochemistry, University of Urbino, 61029 Urbino, Italy; 4 Department of Molecular Medicine & Pathology, University of Auckland, Auckland 1142, Aotearoa, New Zealand; 5 Institute of Cancer Research, London SW3 6JB, UK; 6 Department of Biochemistry, University of Otago, Dunedin 9054, Aotearoa, New Zealand

## Abstract

**Background:**

Inherited genetic factors such as E-cadherin (*CDH1*) promoter variants are believed to influence the risk towards sporadic diffuse gastric cancer (DGC). Recently, a new regulatory region essential for *CDH1 *transcription has been identified in *CDH1 *intron 2.

**Methods:**

We genotyped all known polymorphisms located within conserved sequences of *CDH1 *intron 2 (rs10673765, rs9932686, rs1125557, rs9282650, rs9931853) in an Italian population consisting of 134 DGC cases and 100 healthy controls (55 patient relatives and 45 unrelated, matched individuals). The influence of individual variants on DGC risk was assessed using χ^2^-tests and logistic regression. The relative contribution of alleles was estimated by haplotype analysis.

**Results:**

We observed a significant (p < 0.0004) association of the *CDH1 *163+37235G>A variant (rs1125557) with DGC risk. Odds ratios were 4.55 (95%CI = 2.09–9.93) and 1.38 (95%CI = 0.75–2.55) for AA and GA carriers, respectively. When adjusted for age, sex, smoking status, alcohol intake and *H. pylori *infection, the risk estimates remained largely significant for AA carriers. Haplotype analysis suggested the 163+37235A-allele contributes to disease risk independently of the other variants studied.

**Conclusion:**

The *CDH1 *163+37235G>A polymorphism may represent a novel susceptibility variant for sporadic DGC if confirmed in other populations. Considering the broad expression of E-cadherin in epithelia, this exploratory study encourages further evaluation of the 163+37235A-allele as a susceptibility variant in other carcinomas.

## Background

Gastric cancer is a major cause of cancer-related mortality and is usually classified into two histological types, the intestinal and the diffuse form (Lauren classification [1]). The general incidence rates for stomach cancer are in a steady decline, largely due to decreasing rates of the intestinal cancer-type. This falling frequency is believed to be the result of improved nutrition and sanitary conditions. In contrast, the incidence of diffuse gastric cancer (DGC) alone appears more stable over the past few decades [1,2]. Such a constant rate suggests a larger contribution of inherited genetic risk rather than environmental factors to the diffuse form of stomach cancer.

Owing to its early development underneath the gastric mucosal surface [3], DGC is usually diagnosed at an advanced stage and consequently associated with a poorer outcome [1]. Therefore, genetic DGC markers may facilitate the identification of individuals at risk and thereby contribute to an improvement in DGC diagnosis and therapy.

On a molecular level, DGC is distinguished from the intestinal type on the basis of its abnormal expression of the cell-cell adhesion molecule E-cadherin [4]. E-cadherin is the key component of the epithelial adherens junction and as such is required for functional intercellular adhesion within epithelial sheets [5]. In contrast to many other epithelial cancers, E-cadherin is downregulated very early during DGC development, suggesting a role in the initiation of this disease [3]. Mutation and promoter hypermethylation of the E-cadherin gene (*CDH1*) are the most consistent genetic alterations observed in sporadic DGC [6,7]. Furthermore, *CDH1 *germline mutations predispose to hereditary DGC [8] consistent with an initiating function of E-cadherin deficiency in DGC. *CDH1 *germline mutations usually co-segregate with a dominant pattern of disease among affected families, and occasionally can be found in isolated DGC cases diagnosed at a young age (<45 y) [9]. However, they account for only about 1% of all DGC cases [9] and hence cannot explain the genetic aetiology postulated to contribute to apparent sporadic DGC cases. Genetic alterations other than *CDH1 *germline mutations are therefore likely to add to the risk of developing DGC in the absence of a clear family history or a young age at diagnosis.

Common allelic variants with a mild functional effect can influence the risk for sporadic disease. Indeed, a single nucleotide polymorphism (SNP) within the *CDH1 *promoter (-160C>A) has been associated with a significantly increased risk of sporadic DGC in certain high-incidence populations [10-13]. Of the studied *CDH1 *SNPs, the -160A promoter allele is so far the only variant implicated in DGC risk but appears to act in combination with other *CDH1 *polymorphisms [10,13].

Recently, a new *CDH1 *regulatory region has been described [14]. This region is contained within *CDH1 *intron 2, the largest non-coding *CDH1 *segment (66% of the total sequence) and has been shown to be required for both the initiation and maintenance of transcriptional *CDH1 *activity in differentiated epithelia. Importantly, intron 2 sequences are also necessary for normal *CDH1 *transcription during adult life, providing the possibility that variants within this region may affect diffuse gastric carcinogenesis.

In this study, we genotyped all known variants located within the conserved sequences of *CDH1 *intron 2 and determined their allelic frequencies in groups of Italian sporadic DGC cases and healthy individuals to unravel possible associations with disease.

## Methods

### Patients

DNA samples were obtained from 134 DGC patients who were natives of the District of Pesaro-Urbino, Region Marche, Central Italy. After surgery, the DGC diagnosis was independently confirmed by two pathologists. Patients were clinically evaluated at the local Medical Oncology Unit (Hospital d'Urbino), where they also completed a demographic sheet including their personal and familial cancer history. Data were verified during interviews with their oncology physicians and their family history was traced back for ≥3 generations and laterally to 2^nd ^and 3^rd ^degree relatives. On the basis of this evaluation, none of the patients met the clinical criteria for known familial cancer syndromes. The inclusion criteria for eligible patients were: Caucasian ethnicity, native of the studied geographical area and lack of family history of cancer. The same criteria plus lack of personal history of cancer were adopted for controls. Control DNA samples were obtained from 55 healthy relatives, who were either unaffected parents (n = 15), siblings (22) or children (18) of the studied DGC patients. As healthy relatives were not available for every DGC patient, DNA samples from a group of unrelated healthy individuals (n = 45) identified through the pool of former and current blood donors from the Hospital d'Urbino were included yielding a total of 100 controls. Unrelated controls were randomly selected with frequencies matching to cases by age and sex. The mean age of DGC patients without relatives was 54.6 y ± 11.41SD, while that of their matched controls was 52.2 y ± 10.21SD. All subjects were interviewed about their smoking and drinking habits. *H. pylori *status was determined by pathological examination of gastric samples for cases, and by blood or breath tests for controls. The ethical requirements were verified and approved by the internal Ethical Committee (Hospital d'Urbino) and all study participants gave their written informed consent.

### CDH1 intron 2 conserved regions and polymorphisms

Conserved regions of *CDH1 *intron 2 (GenBank NC_000016) were identified by retrieving corresponding human, chimpanzee, rat and mouse sequences from the NCBI database (NCBI, Entrez nucleotide) followed by alignment using the NCBI server (NCBI, Basic Local Alignment Search Tool) and Invitrogen Vector NTI Advance™ 9.0 software (Accelrys Software Inc, San Diego, USA). Conserved regions were defined as having less than 5% sequence variations among the different species. The conserved regions were PCR-amplified into overlapping fragments of about 200 bp size. The corresponding primers (see Table 1 for sequences and conditions) were designed using the GeneFisher online tool [15] and manufactured by Sigma-Proligo (Sigma-Aldrich Corporation, St. Louis, USA). FastStart Taq DNA Polymerase (Roche, Basel, Switzerland) and PTC-200 PCR machines (MJ Research, Waltham, USA) were used. The following polymorphisms are located (Ensemble GenomeBrowser [16]) within the amplified regions: 163+14184ΔAGGG (rs10673765, located in PCR fragment C2F1), 163+14384C>T (rs9932686, C2F2), 163+37235G>A (rs1125557, C3F2), 163+37276T>A (rs9282650, C3F2), and 163+49526C>G (rs9931853, C4F1). The TESS online tool [17] was used to search for putative transcription binding factor sites that may be affected by the above variants.

**Table 1:** PCR primers and conditions

	**Forward primer**	**Reverse primer**	T_a_*	**Mg^++†^**	**DMSO^‡^**
**C1F1**	ccgccttaaagaaactcttg	accggtggcaaatactag	65°C	1.5	-
**C1F2**	tagaagggttgaacctgttc	tcttagtccacgagaagaag	65°C	1.5	-
**C1F3**	taggagagcttgtaacaagc	cactcggttctaccgaag	65°C	1.5	-
**C2F1**	tgtattagccacagagaag	ctaaaactagaccacgaag	65°C	1.5	-
**C2F2**	gtcacaaaacagcttg	ccttccttgagcaaggc	65°C	1.5	-
**C3F1**	ttgcctaaggccccctttttgttc	gaatctgcgaagtctacatc	65°C	1.5	-
**C3F2**	acactagccacacatgggactcaag	tgctggtgtggattcaaatgtg	65°C	1.5	-
**C4F1**	acctccgcctcctgggttcaagc	ttcctcccgcttagtg	60°C	1.5	-
**C4F2**	tggccaggcctgtcttaaactc	ttcttaggtccgtgggtttttacg	65°C	1.5	-
**C4F3**	aaagtgctgggattacaggtgtgag	tcgataatcccgagaactc	55°C	1.0	**+**
**C4F4**	gaaccataggactttgactgatgg	actgatggttatccgggttcccttg	65°C	1.5	-
**C4F5**	agctgttgagctgtcatcacaatcc	gaatttcctacccgtctatggtagg	65°C	1.5	-
**C5F1**	tagtggggagtggggtcttagcttc	tcgttcaccctcctttcttcttacc	58°C	1.5	-
**C5F2**	gggcatgttgaaatatacccagtc	tctgagtaatagaggggtacgttgg	65°C	1.5	-
**C5F3**	cttgccagcgtgacagtg	cgaaaccccgtggagtag	65°C	1.5	-
**C5F4**	caggttggggctcctcgtcatactg	cttccgacgtgacttaaggaaagag	65°C	1.5	-
**C5F5**	gcttgtctcaactttcactgtc	gaatttcctacccgtctatggtagg	65°C	1.5	-
**C6F1**	tggtattcaggaggatgcag	acctacgatcgtaaaaagt	65°C	1.5	-
**C6F2**	cccatcaatgcttatttgttctt	gcctgggagacggagact	65°C	1.5	-
**C6F3**	tgggctgtttgagttttgttc	cggtgtaaaaggttcgtgac	65°C	1.5	-

*T_a _annealing temperature; ^†^Mg^++^-concentration is given in mM; ^‡^DMSO was added at 5% f.c.

### Single-strand conformation polymorphism

Single-strand conformation polymorphism (SSCP) was used to scan the conserved intron 2 region in 19 Italian DGC patients for the presence of additional common but population-specific polymorphisms. SSCP was performed as described [18], with the exception that ULS™ 495 fluorophore (Kreatech Biotechnology, Amsterdam, Netherlands) was used instead of radioactivity to label the fragments. In brief, 1 μl PCR product was incubated with 0.2 μl dye in a 20 μl reaction. Gels were scanned using an FX molecular imager (BioRad, Hercules, USA) at 488 nm.

### Genotyping

The following restriction enzymes were used for the genotyping of DNA variants: 0.06 U/μl BsaXI for 163+14184ΔAGGG, 1 U/μl *BanII *for 163+14384C>T, 0.2 U/μl *MaeIII *for 163+37276T>A, and 0.4 U/μl *HpaII *for 163+49526C>G. All enzymes were from New England Biolabs (Ipswich, USA) with the exception of *MaeIII *from Roche (Basel, Switzerland). Reactions were incubated overnight and fragments were separated on 4%(w/v) agarose gels.

Polymorphisms 163+37235G>A and 163+37276T>A were genotyped on a ABI Prism 7900 (Applied Biosystems, Foster City, USA) using the real-time PCR-based allelic discrimination assays from Applied Biosystems according to the instructions provided.

### Sequencing

Detected variants were verified by direct sequencing using the USB thermosequencing kit (USB, Cleveland, USA) and a LiCor 4000L DNA sequencer (LiCor, Lincoln, Nebraska USA).

### Statistical analysis

Differential distributions among cases and controls were assessed by the χ^2^-test (with df = 2 for genotypes and df = 1 for alleles). Risk was estimated by univariate analysis and by multiple logistic regression (STATA software, StataCorp LP, College Station, USA). The χ^2^-test (df = 2) was also used to examine deviations from Hardy-Weinberg equilibrium. Age differences among patients carrying different genotypes were calculated using a 2-tailed t-test.

Haplotype frequencies were reconstructed from unphased genotypes and linkage disequilibrium (LD) between SNPs was estimated using the SHEsis software platform [19,20]. Only haplotypes with a relative frequency >0.03 in either cases or controls were included in the analysis. Global association of haplotypes with disease was calculated by a χ^2^-test (df = 7). The 163+14184ΔAGGG and 163+14384C>T variants were not included into the final analysis as they were not informative. The association of individual haplotypes with disease was based on 2 × 2 contingency tables in comparison to the A-A-C haplotype. LD was expressed as r^2^, with r^2 ^= 1 indicating complete LD, r^2 ^= 0 absence of LD, and r^2 ^< 0.33 suggesting minimal LD.

## Results

Six conserved regions with a total size of 3.2 kbp were identified within *CDH1 *intron 2. Apart from the five known polymorphisms (*CDH1*163+14184ΔAGGG (rs10673765), 163+14384C>T (rs9932686), 163+37235G>A (rs1125557), 163+37276T>A (rs9282650), and 163+49526C>G (rs9931853)), no additional common polymorphisms specific for the Italian population under study were discovered by SSCP in the six regions.

Using restriction fragment length polymorphism and allelic discrimination assays, the relative frequencies of the genotypes resulting from the five variants were determined in the DGC cases and the controls. Sequencing of random samples confirmed the respective genotypes. All polymorphisms were in Hardy-Weinberg equilibrium for both cases and controls (p > 0.19). Table 2 summarises the genotype distributions and their differences between cases and controls.

**Table 2:** *CDH1 *intron 2 genotype distributions among DGC cases and controls

**+14184ΔAGGG cases (n = 134)**			**+14184ΔAGGG controls (n = 100)**			**χ^2^-test**	OR (95%CI)^*,†^	OR (95%CI)^*,†^
**+/+**	**+/Δ**	**Δ/Δ**	**+/+**	**+/Δ**	**Δ/Δ**	**p**	**Δ/Δ vs +/+**	**+/Δ vs +/+**
128	4	2	96	3	1	0.947	1.50 (0.13–16.78)	1.00 (0.21–4.57)
95.5%	3%	1.5%	97%	1.5%	1.5%		14.2%	2.5%

**+14384C>T cases (n = 134)**			**+14384C>T controls (n = 100)**			**χ^2^-test**	**OR (95%CI)**	**OR (95%CI)**

**CC**	**CT**	**TT**	**CC**	**CT**	**TT**	**p**	**TT vs CC**	**CT vs CC**
130	2	2	98	1	1	0.895	1.51 (0.13–16.87)	1.51 (0.13–16.87)
97.0%	1.5%	1.5%	98.0%	1.0%	1.0%		12.3%	12.3%

**+37235G>A cases (n = 134)**			**+37235G>A controls (n = 100)**			**χ^2^-test**	**OR (95%CI)**	**OR (95%CI)**

**GG**	**GA**	**AA**	**GG**	**GA**	**AA**	**p**	**AA vs GG**	**GA vs GG**
30	56	48	37	50	13	0.0003	4.55 (2.09–9.93)	1.38 (0.75–2.55)
22.4%	41.8%	35.8%	37.0%	50.0%	13.0%		100%	40.6%

**+37276T>A cases (n = 134)**			**+37276T>A controls (n = 100)**			**χ^2^-test**	**OR (95%CI)**	**OR (95%CI)**

**TT**	**TA**	**AA**	**TT**	**TA**	**AA**	**p**	**AA vs TT**	**TA vs TT**
65	65	4	46	51	3	0.929	0.94 (0.20–4.42)	0.90 (0.53–1.53)
48.5%	48.5%	3.0%	46.0%	51.0%	3.0%		1.9%	1.6%

**49526C>G cases (n = 134)**			**+49526C>G controls (n = 100)**			**χ^2^-test**	**OR (95%CI)**	**OR (95%CI)**

**CC**	**CG**	**GG**	**CC**	**CG**	**GG**	**p**	**GG vs CC**	**CG vs GG**
34	82	18	30	58	12	0.727	1.32 (0.55–3.19)	1.25 (0.69–2.26)
25.4%	61.2%	13.4%	30.0%	58.0%	12.0%		32.2%	22.5%

*OR values are unadjusted.

^†^The percentage below the OR values estimates the power of association for each variant at the corresponding OR level and assuming a significance level of 5%.

Of the investigated variants, only the 163+37235G>A SNP was significantly associated with disease due to an overrepresentation of the A-allele among the DGC cases (56.7% vs. 38% in controls, χ^2 ^= 16.1, p < 0.0001; see Table 2). The 163+37235AA genotype was 2.8 times more frequent in cases compared to controls. The corresponding Odds Ratio (OR) suggested a significantly elevated risk of developing DGC for AA carriers relative to GG carriers (OR = 4.55, 95%CI = 2.09–9.93, p = 0.0002, power of association 100%; Table 2). No significant increase in risk was apparent from carrying the GA-genotype (OR = 1.38, 95%CI = 0.75–2.55, p = 0.3, power of association 41%; Table 2). The DGC risk for AA carriers remained significant, when ORs were adjusted for age, sex, alcohol intake and *H. pylori *infection (Table 3). In smokers, however, the associated risk was only of borderline significance (p = 0.089, Table 3). The risks associated with the other variants studied remained non-significant following adjustment (data not shown). No association was observed between the 163+37235G>A SNP and age at diagnosis (p > 0.16).

**Table 3:** Adjusted ORs associated with the *CDH1 *intron 2 163+37235G>A variant

**Variable**		**163+37235**	**Cases**		**Controls**		**OR (95% CI)**
					
			**n**	**%**	**n**	**%**	
**Age**	**≤ Median**	**GG**	17	20	20	34	1
		**GA**	37	45	30	52	1.45 (0.65–3.25)
		**AA**	29	35	8	14	4.26 (1.55–11.77)
	**> Median**	**GG**	13	25	17	40	1
		**GA**	19	37	20	48	1.24 (0.48–3.24)
		**AA**	19	37	5	12	4.97 (1.47–16.86)

**Sex**	**Female**	**GG**	13	20	20	34	1
		**GA**	28	44	30	51	1.44 (0.60–3.42)
		**AA**	23	36	9	15	3.93 (1.39–11.12)
	**Male**	**GG**	17	24	17	41	1
		**GA**	28	40	20	49	1.40 (0.58–3.39)
		**AA**	25	36	4	10	6.25 (1.79–21.84)

**Smoking**	**Never**	**GG**	17	24	25	42	1
		**GA**	30	42	30	50	1.47 (0.66–3.26)
		**AA**	25	35	5	8	7.35 (2.34–23.01)
	**Ever**	**GG**	13	21	12	30	1
		**GA**	26	42	20	50	1.20 (0.45–3.19)
		**AA**	23	37	8	20	2.65 (0.86–8.16))

**Alcohol**	**≤ 20 g/day**	**GG**	24	26	26	40	1
		**GA**	35	38	31	48	1.22 (0.59–2.55)
		**AA**	34	37	8	12	4.60 (1.78–11.90)
	**> 20 g/day**	**GG**	6	15	11	31	1
		**GA**	21	51	19	54	2.01 (0.63–6.55)
		**AA**	14	34	5	15	5.13 (1.23–21.36)

***H. pylori***	**Negative**	**GG**	2	11	16	37	1
		**GA**	22	48	22	51	3.2 (1.00–10.26)
		**AA**	19	41	5	12	12.16 (2.98–49.64)
	**Positive**	**GG**	25	28	21	37	1
		**GA**	34	39	28	49	1.02 (0.472–-.19)
		**AA**	29	33	8	14	3.045 (1.15–8.07)

To determine whether the *CDH1 *163+37235A-allele confers DGC risk independently or in combination with the other intron 2 variants, haplotypes resulting from the five polymorphisms were reconstructed and their frequencies were estimated in the cases and controls. The two 5'-variants were not informative and were thus excluded. The intron 2 haplotypes showed a global association with disease (df = 7, χ^2 ^= 24.09, p < 0.002). In general, haplotypes containing the 163+37235A-allele were more frequent in cases compared to controls, while three of the four haplotypes with the G-allele were more frequent among the controls (Table 4). The strongest association with disease was observed for the AAG and the ATC haplotypes (with 163+37235A at position 1). Conversely, the GAG and the GTC haplotypes showed the strongest protection. Linkage disequilibrium analysis indicated largely absent LD between the investigated variants (r^2 ^< 0.03; Table 5), suggesting that the *CDH1 *163+37235G>A SNP may confer an increased susceptibility towards DGC independently of the other variants investigated.

**Table 4:** *CDH1 *intron 2 haplotype frequencies among DGA cases and controls

	**Case (freq)^†^**	**Control (freq)^†^**	**Fisher's *p***	**Pearson's *p***	**OR (95%CI)**
AAC*	16.04 (0.060)	5.31 (0.027)	0.088	0.088	2.33 (0.86–6.34)
**AAG**	17.49 (0.065)	5.34 (0.027)	0.056	0.056	2.55 (0.95–6.83)
**ATC**	73.09 (0.273)	35.43 (0.177)	0.015	0.015	1.74 (1.11–2.74)
**ATG**	45.38 (0.169)	29.91 (0.150)	0.565	0.565	1.16 (0.70–1.92)
**GAC**	21.73 (0.081)	24.18 (0.121)	0.152	0.152	0.64 (0.35–1.18)
**GAG**	17.75 (0.066)	22.17 (0.111)	0.087	0.087	0.57 (0.30–1.09)
**GTC**	39.15 (0.146)	53.07 (0.265)	0.001	0.001	0.47 (0.30–0.75)
**GTG**	37.37 (0.139)	24.85 (0.123)	0.602	0.602	1.16 (0.67–1.99)

*Haplotype order: 163+37235G>A, 163+37276T>A, 163+49526C>G. The two 5'-variants were not included, as they did not further split up the haplotypes.

^†^Numbers refer to reconstructed haplotype numbers among cases (257) and controls (192), with each individual carrying two chromosomes. Relative frequencies are given in percent. Not all genotype data was included into analysis, as low frequency haplotypes were dropped.

**Table 5:** Linkage disequilibrium between *CDH1 *intron 2 polymorphisms

**r^2 ^linkage disequilibrium**				
	**163+14384C>T**	**163+37235G>A**	**163+37276T>A**	**163+49526C>G**

**163+14184ΔAGGG**	0.001	0.000	0.001	0.001
**163+14384C>T**	-	0.002	0.000	0.000
**163+37235G>A**	-	-	0.028	0.000
**163+37276T>A**	-	-	-	0.003

Eighteen of the healthy controls related to the DGC patients were children. A few of them may develop DGC later in their life. We therefore repeated the analysis excluding all 18 children from the controls. The associations between the 163+37235A-containing genotypes/haplotypes with disease were similar to those obtained without exclusion. As expected, however, all associations were more significant and corresponding risk estimates increased (data not shown).

## Discussion

Inherited genetic risk is believed to be a crucial factor contributing to the incidence of sporadic DGC. Little is known, however, about susceptibility loci that may confer DGC risk without evoking an apparent family history. *CDH1*, coding for the epithelial adhesion molecule E-cadherin, is one of the few known genes to have an etiologic role in DGC. So far, only one polymorphism located within the *CDH1 *promoter has been implicated in the sporadic DGC risk of certain populations [10-13]. In this study, we sought to assess a possible contribution of *CDH1 *intron 2 variants to disease risk, as this region has recently been shown to be essential for normal *CDH1 *transcription during adult life, similar to the *CDH1 *promoter.

Our present results suggest a role of *CDH1 *intron 2 alleles in the risk of developing sporadic DGC and identify the 163+37235G>A SNP as a putative susceptibility variant. Both individual genotype data and corresponding haplotype data are consistent with a contribution of the *CDH1 *163+37235A-allele to DGC risk that is independent of the other four investigated *CDH1 *intron 2 variants.

A strength of our study is the inclusion of healthy relatives into the control group. Unaffected relatives are expected to share more genetic variants with their related patients compared to unrelated, matched controls, resulting in a reduction of background genetic noise. While the genetic relation may decrease the significance level of an association, the detected genetic differences are likely more robust. Consistent with this, exclusion of relatives too young to have disease strengthened the association of the 163+37235A-allele with DGC. For all controls, the ratio of the relative frequencies of the A- and G-alleles (A:G) was 0.613, which is higher than reported for other Caucasian populations (0.38, GenomeBrowser [16]). However, the A:G ratio for unrelated controls only was 0.215, suggesting that the higher ratio is due to an enrichment of the disease allele among the relatives of DGC patients as one would expect for a high risk population. Somewhat unusual was the lack of any significant LD among the SNPs investigated. Intermarker values between the 163+37235G>A and 163+37276T>A variants were available for other populations on the HapMap Browser [21]. Strong LD was also absent in four different ethnic groups (r^2 ^range = 0.12–0.52), suggesting independent segregation of these variants may be common. Together, the data are consistent with a direct association of the 163+37235A-allele with an increased susceptibility to DGC.

A limitation of this study is its small sample size. The study was designed to detect at the 5% level of significance an OR of 2.0 for relatively common variants with power >90%. Thus, the observed lack of significant associations may well be due to inadequate power to detect variants of weaker effect, particularly with rarer alleles. Larger sample sizes will be required to conclusively assess the impact of these variants on disease risk. However, our aim was to identify common susceptibility loci that confer a relatively strong risk and hence might contribute to a significant number of apparent sporadic DGC cases. Another potential limiting factor is that the observed association is due to LD with other disease variants not investigated here. A candidate variant may be the *CDH1 *-160A-allele. Genotype data for the -160 SNP were available for a subgroup of the cases studied. Preliminary analysis suggested absence of LD between the 163+37235 and the -160 SNP (r^2 ^= 0.008; BH, unpublished results), consistent with an independent contribution of the intron 2 variant to disease. This idea is supported by the consistent association of 163+37235A-containing haplotypes with DGC (Table 4). Additionally, the observation that in mice the promoter and intron 2 are both independently required for *CDH1 *activity [14] is further evidence for an autonomous role of the intron 2 variant in DGC susceptibility. Moreover, the risk associated with the intron 2 variant remained significant following the adjustment for potential confounding factors, in support of a direct association. An exception was smoking, which reduced the associated risk to borderline significance. Of note, the risk estimates increased in both non-smokers and *H. pylori*-negative patients. While these trends may be due to the relatively low numbers of subjects in stratified subgroups, they may be consistent with genetic DGC risk factors being more important in individuals not exposed to environmental risks.

*CDH1 *intron 2 sequences are vital both to initiate transcriptional activity and to maintain E-cadherin expression in differentiated epithelia of mice [14]. Given the conserved function of E-cadherin in different species, it is very probable that intron 2 is also essential for *CDH1 *activity in human epithelia. This is supported by the presence of highly conserved regions within intron 2, suggesting that the conserved elements may be binding sites for transcription factors participating in *CDH1 *regulation. A search for putative binding sites revealed that the *CDH1 *163+37235 position lies within a recognition motif of the human nuclear factor I/X (NFIX, *OMIM *#164005). According to the TESS web page [17], the 163+37235G position (TGGCA) is the most conserved nucleotide within the NFIX recognition sequence, suggesting the 163+37235A-allele may alter the affinity of this transcription factor to the cis-regulatory element. Whether NFIX indeed is able to regulate E-cadherin expression in gastric tissue remains to be determined. In mice, however, NFIX is essential for embryonic development [22] and is expressed in many adult tissues including epithelial ones [23]. Genes regulated by NFIX have been identified and include repression of pro-angiogenic PDGFA [24] and p21, where repression surprisingly leads to growth inhibition [25]. NFIX may also slow down cell growth via downregulation of the adenine nucleotide translocase-2 gene (*ANT2*) [26]. In addition, NFIX has been shown to confer resistance towards transformation by nuclear but not cytoplasmic oncogenes [27]. The reported findings are compatible with a tumour suppressing role of NFIX, where reduced binding of this transcription factor to its recognition sequence may favour tumorigenic events. It is thus of some interest that the chromosomal region 19p13.3, the location of *NFIX*, appears frequently deleted in some [28,29], however not all [30] series of gastric cancers.

To our knowledge, this is the first study to report an association between an intronic *CDH1 *variant and sporadic cancer. Similar studies will be required to confirm an association with DGC in other populations/ethnicities and, using a larger sample size, to determine whether the 163+37235A variant may also be a disease allele in cohorts from low DGC risk regions. Furthermore, case-control studies on other carcinoma types, including other gastric cancer histotypes, should address whether the intron 2 variant is specifically associated with DGC. Regarding the *CDH1 *promoter SNPs, the -160A-allele has not only been implicated in DGC in Italians [10,11], but also in a Mexican [12] and a Japanese [13] population (here included in disease haplotypes). Not all reports have found significant associations, suggesting population-specific effects or differences in study design [31,32]. To address this heterogeneity, Wang *et al*. have evaluated data from 11 case-control series and concluded that the -160A-allele is a gastric cancer susceptibility allele in European, but not Asian populations [33]. However, their meta-analysis did not include very recent studies reporting positive associations [11-13] and therefore may underestimate the contribution of the -160A-allele to gastric cancer. Further positive associations have been observed with sporadic carcinomas at other sites such as prostate [34,35], urether [36], bladder [37], breast [38], colorectum, and endometrium [11]. Another *CDH1 *promoter variant, -347insA, has not been associated with sporadic DGC so far, but appears to increase susceptibility to colorectal cancer [39] and to contribute to oesophageal/cardiac cancer risk [40]. Such observations imply that functional *CDH1 *variants may be involved in the susceptibility towards a broad range of epithelial cancers, consistent with the expression pattern of E-cadherin in tissues. It appears likely that, in many instances, *CDH1 *variants will require the presence of other etiologic factors to increase disease risk, as E-cadherin deficiency is usually associated with cancer progression rather than initiation [41]. Furthermore, *CDH1 *polymorphisms might also affect the differentiation degree of tumours; preliminary results from our laboratory suggest an overrepresentation of specific *CDH1 *haplotypes in poorly differentiated lung carcinomas (Emily Wilson, HSc-thesis). Therefore, the role of the intronic *CDH1 *163+37235A variant as a disease allele may not be limited to sporadic DGC risk.

## Conclusion

We report the identification of a new putative susceptibility variant for DGC located within conserved sequences of *CDH1 *regulatory intron 2. Both individual genotype and haplotype data suggest a contribution of the *CDH1 *163+37235A-allele to sporadic DGC risk that is independent of other *CDH1 *variants within conserved intron 2 regions. A larger confirmatory study involving other populations and including complete *CDH1 *haplotypes will be required to assess the population-specificity and the relative contribution of the 163+37235A-allele to disease. The establishment of heritable DGC risk factors will be helpful particularly with respect to the difficult and often delayed diagnosis of diffuse-type stomach cancer. Given the universal expression of E-cadherin in epithelial tissues, this exploratory study may also provide a basis to investigate the role of the *CDH1 *163+37235G>A SNP in the incidence and progression of tumours other than DGC.

## Competing interests

The authors declare that they have no competing interests.

## Authors' contributions

SN performed the SSCP screen, the genotyping, analysed the data, and helped to draft the manuscript. HM identified the conserved sequences, designed the PCR primers, the PCR reactions and the genotyping assays. FG organised the sample collection and patient data, participated in the collection of blood, performed case/control interviews and helped to design the study. AR participated in the sample collection and isolated the DNA. EW participated in the genotyping and in the data analysis. AD helped to analyse the data and to design the study. CM helped to analyse the data. TM helped to analyse the data and to draft the manuscript. PG helped to design the study and revised the manuscript. MM helped to organise samples and to design the study. BH conceived of the study, helped to analyse the data and drafted the manuscript. All authors read and approved the final manuscript.

## Pre-publication history

The pre-publication history for this paper can be accessed here:


